# Chemogenetic inhibition of the lateral hypothalamus effectively reduces food intake in rats in a translational proof-of-concept study

**DOI:** 10.1038/s41598-024-62014-1

**Published:** 2024-05-18

**Authors:** Péter Kovács, Tamás Kitka, Zsolt Kristóf Bali, Lili Veronika Nagy, Angelika Bodó, Tamás Kovács-Öller, Zalán Péterfi, István Hernádi

**Affiliations:** 1 VRG Therapeutics, Füvészkert utca 3., Budapest, 1083 Hungary; 2 Grastyán Endre Translational Research Centre, University of Pécs, 6 Ifjúság str., Pécs, 7624 Hungary; 3 Translational Neuroscience Research Group, Centre for Neuroscience, Szentágothai Research Centre, University of Pécs, 20 Ifjúság str., Pécs, 7624 Hungary; 4 Department of Neurobiology, Faculty of Sciences, University of Pécs, 6 Ifjúság str., Pécs, 7624 Hungary; 5 Histology and Light Microscopy Core Facility, Szentágothai Research Centre, University of Pécs, 20 Ifjúság str., Pécs, 7624 Hungary; 6 Institute of Physiology, Medical School, University of Pécs, 12 Szigeti út, Pécs, 7624 Hungary

**Keywords:** DREADD, Obesity, Food-intake management, Body weight, Deschloroclozapine, Clozapine-*N*-oxide, Gene delivery, Genetic engineering, Feeding behaviour, Genetics of the nervous system

## Abstract

Despite the therapeutic potential of chemogenetics, the method lacks comprehensive preclinical validation, hindering its progression to human clinical trials. We aimed to validate a robust but simple in vivo efficacy assay in rats which could support chemogenetic drug discovery by providing a quick, simple and reliable animal model. Key methodological parameters such as adeno-associated virus (AAV) serotype, actuator drug, dose, and application routes were investigated by measuring the food-intake-reducing effect of chemogenetic inhibition of the lateral hypothalamus (LH) by hM4D(Gi) designer receptor stimulation. Subcutaneous deschloroclozapine in rats transfected with AAV9 resulted in a substantial reduction of food-intake, comparable to the efficacy of exenatide. We estimated that the effect of deschloroclozapine lasts 1–3 h post-administration. AAV5, oral administration of deschloroclozapine, and clozapine-*N*-oxide were also effective but with slightly less potency. The strongest effect on food-intake occurred within the first 30 min after re-feeding, suggesting this as the optimal experimental endpoint. This study demonstrates that general chemogenetic silencing of the LH can be utilized as an optimal, fast and reliable in vivo experimental model for conducting preclinical proof-of-concept studies in order to validate the in vivo effectiveness of novel chemogenetic treatments. We also hypothesize based on our results that universal LH silencing with existing and human translatable genetic neuroengineering techniques might be a viable strategy to affect food intake and influence obesity.

## Introduction

Chemogenetics is an emerging gene therapy method, by which genetically “designed” new receptors are expressed on specific neurons in the central nervous system (CNS), after a viral transfection^1–3^. Subsequently, by peripherally applying low doses of otherwise biologically inactive small molecule drugs (“actuators”), specific brain areas or neuronal networks can be “turned on or off”, on demand. This developing neuroscience method is not only providing a unique research tool for neuroscientists^4^ but also has the potential to open a new class of novel CNS medicines in the near future^3,5,6^, offering a superior therapeutic alternative to current pharmacotherapy and deep brain stimulation (DBS) methods, in several neurological and psychiatric diseases, including obesity.

One of the main reasons why—despite their very promising therapeutic potential—chemogenetic methods have not yet reached human clinical research is the fact that they lack proper preclinical validation including the optimization of several important experimental and methodological factors. For example, one typical validity issue in the available animal studies is the general use of the actuator molecule clozapine-*N*-oxide (CNO) to stimulate chemogenetic receptors, which, surprisingly, turned out to be hardly brain penetrant, non-selective, and metabolically unstable, therefore it is not ideal for the specific activation of chemogenetic receptors in the brain^7,8^. Other gaps in our knowledge include the lack of sufficient proof for the long-term in vivo efficacy and safety of the chemogenetic approach, the pharmacodynamic and pharmacokinetic optimization of appropriate actuator molecules (i.e., deschloroclozapine, DCZ), the optimization of the generally applied Adeno-Associated Virus (AAV) gene transfer method (virus dose, titer, promoter and serotype selection), the proper toxicological evaluation in the nervous tissue, and the validation of appropriate proof-of-concept (POC) and “first in vivo” methods to measure in vivo effectiveness quickly and reliably in rodent behavioral paradigms. Although the generation of in vivo chemogenetic POC studies has been started (e.g., in Parkinson's disease^9–11^ or epilepsy^12–14^), the human translational validity of these pioneering studies is debatable, because of the above detailed methodological flaws. Moreover, several high unmet need CNS indications would be potentially suitable targets for chemogenetic drug discovery^2^, and more that are completely lacking the proper preclinical in vivo POC studies, including obesity, as a potential future target indication for chemogenetic therapies.

There is a general hypothesis in the literature that presumes that one of the easiest ways to develop new chemogenetic therapies is to modify the existing DBS neurosurgical technology and leverage the fact that the surgical intervention that is applied in currently approved DBS methods is more or less the same as a chemogenetic application would require^3^. Moreover, chemogenetic surgical techniques might provide a safer option to patients because, in contrast to DBS, they do not require chronically implanted devices with the risk of developing postoperative complications^15^.

If we take a closer look at the potential anti-obesity applications of future chemogenetic human therapies, there are encouraging human DBS results in the literature, and clinical trials, including the lateral hypothalamus (LH), as a target brain structure^16,17^. A considerable amount of case studies that utilized hypothalamic DBS targets resulted in weight loss in obese patients^16^. Therefore, we hypothesize that a less risky surgical method (compared to DBS) accompanied by a potentially more effective (chemogenetic) modulation of LH or other obesity-related brain circuits might provide a new therapeutic alternative to severely obese patients next to pharmacotherapy and bariatric surgery.

There are several rodent studies in the literature that demonstrate the potential effectiveness of chemogenetic methods in reducing food intake^5,18–21^, however none of the previous studies utilized methods that would be directly translatable to human therapeutic applications. Some of these promising previous chemogenetic studies validated the potential of LH^20,21^ or arcuate nucleus^18,19^ genetic modification leading to the effective modulation of food intake, however, all of the previous studies utilized non human-translatable methods, for example ‘Cre-lox’ techniques, that can successfully be applied in animal models, however they do not possess any human translational value what could be readily translated into novel therapeutical interventions. Therefore, our aim in the current study was to describe a simple, however high translational value chemogenetic POC to support the development of potential future chemogenetic therapies to be applied in obesity indication in humans, by effectively reducing hunger and food intake without the usually observed post-drug rebound effects. Moreover, our additional goal was to aid the development of new chemogenetic systems towards human use, in general, by describing a simple but robust rodent behavioral assay by which the in vivo effectiveness and basic pharmacokinetic-pharmacodynamic properties of CNS-targeted chemogenetic treatments can be easily and reliably tested. We believe that the described method can serve as a “first in vivo” pharmacodynamic tool to aid early chemogenetic drug discovery, in any CNS indications.

## Materials and methods

### Animals

Young male Lister hooded rats (Envigo, Horst, Netherlands) weighing 320 to 360 g at surgery were pair-housed under a 12/12 h daily light/dark cycle with controlled temperature and humidity in the animal house of the Department of Neurobiology, Faculty of Sciences, University of Pécs, Hungary. Water and food (Ssniff Rat/Mouse Maintenance chow, ssniff Spezialdiäten GmbH, Soest, Germany) were available ad libitum except for the day before food intake experiments. The experiments were approved by the Animal Welfare Committee of the University of Pécs, and the National Scientific Ethical Committee on Animal Experimentation (ÁTET) at the Ministry of Agriculture (ethical license no.: BA02/2000–40/2021). All procedures fully complied with Decree No. 40/2013. (II. 14.) of the Hungarian Government and the EU Directive 2010/63/EU on the Protection of Animals Used for Scientific Purposes. The ARRIVE Guidelines were taken into account in the reporting of the study.

### Intracranial injection of AAVs

The rat was anesthetized with a combination of ketamine (100 mg/kg, intraperitoneal, i.p.) and xylazine (25 mg/kg, i.p.) and was placed in a stereotaxic frame. An incision was made in the skin of the head and soft tissues were eliminated to expose the skull. The skull was washed two times with hydrogen peroxide, then holes were drilled in the skull bilaterally over the LH. A Hamilton (Reno, NV) microsyringe was filled with PBS or a formulation of viral vectors (Addgene, Watertown, MA) carrying sequences of the human synapsin promoter, the designer receptor exclusively activated by designer drug (DREADD, modified human muscarinic receptor 4, hM4D(Gi)), and the mCherry reporter gene. Two different serotypes were used in separate groups of animals: serotype AAV5 (AAV5- hSyn-hM4D(Gi)-mCherry, cat. no. 50475-AAV5) was applied at 7 × 10^12^ vg/ml in n = 4 rats and AAV9 (AAV9- hSyn-hM4D(Gi)-mCherry, cat. no. 50475-AAV9) was applied at 1 × 10^13^ vg/ml in n = 4 other rats. A group of animals (n = 4) received an infusion with PBS into the LH as a control group. The microsyringes were inserted in a Stoelting Quintessential Stereotaxic injector (Wood Dale, IL) and were introduced into the brain to the stereotaxic coordinates AP: − 3.0, ML: 1.8, DV: 8.3 mm from Bregma according to the rat stereotaxic atlas of Paxinos and Watson^22^. After the correct positioning of the microsyringe, 1000 nl of the viral formulation was infused at 100 nl/min speed. After the end of the infusion, we waited an additional 10 min, then the microsyringe was slowly removed from the brain. Injections were made bilaterally into the LH. After injections, the skin of the head was closed using stainless steel wound clips. The weight, food and water intake, and general well-being of the rats were monitored daily after the surgery for 2 weeks. The first food intake experiment started 7 weeks after the surgery.

### Experimental design

The first experiment was designed to validate our food intake measurement protocol using exenatide, a potent GLP-1 agonist compound known for its inhibitory effect on food intake^23,24^. Exenatide was administered to naïve (non-AAV-injected) rats as a pharmaceutical formulation (Byetta, Eli Lilly, Netherlands) at doses of 2.5, 10, and 50 µg/kg s.c. The effects of exenatide were compared to its vehicle which was 0.1 N acetate buffer (pH = 5.0). Further experiments were designed to demonstrate the effects of chemogenetic silencing of the LH on the food intake of the animals that were previously intracranially injected with DREADD-carrying AAV. First, we tested the effects of subcutaneously (s.c.) applied CNO at doses of 100, 300, and 1000 µg/kg. Next, another DREADD actuator, DCZ was tested in the food intake experiments at doses of 30, 100, and 300 µg/kg (s.c.). The vehicle of CNO and DCZ was 0.01 M phosphate-buffered saline (pH = 7.4). Later, DCZ was also administered via oral route (gavage) at doses of 100, 300, and 1000 µg/kg. Subcutaneous injections were applied 30 min prior to testing (re-feeding), while oral drug administrations were applied 60 min prior to re-feeding. However, in an additional experiment, we also tested the time window of the effects of DCZ on the food intake of the animals. Thus, we administered 100 µg/kg DCZ (s.c.) to animals 0.5, 1, 3, or 16 h prior to re-feeding. All food intake experiments were performed in a within-subject design thus, the different treatments (doses or pre-treatment times) were applied to all animals on different days according to a counterbalanced Latin-square design.

### Food intake measurements

Food was taken away from the rats 16 h prior to the start of the food intake experiments to achieve a similar level of hunger in the animals. At the start of the experiment, animals were re-fed with 100 g of the same laboratory chow used as a general maintenance food. The residual amount of laboratory chow was first measured 30 min after re-feeding. Then, the second measurement was performed at 1 h after re-feeding, and further measurements were taken every hour until 8 h after re-feeding. One exception was the experiment assessing the effects of CNO in which the food intake was not measured between 3 and 8 h after re-feeding. The cumulative food intake of the animals in a given measurement time point was calculated as 100 g minus the residual amount of food measured in their feeder in a given time point.

### Histology

At the end of the project, rats were sacrificed by the administration of an overdose of pentobarbital anesthetic (800 mg/kg, i.p.). Brains were rapidly removed and immersed into 4% paraformaldehyde (Sigma) solution for a few days. Then, the brains were put into 30% sucrose (Sigma) solution until they sank to the bottom of the containers indicating that the brains were fully saturated with sucrose. Then, the brains were sectioned using a freezing microtome (Leica, Wetzlar, Germany), and sections were mounted on microscope slides and covered using Vectashield with DAPI (Vector Laboratories, Newark, CA). The slides were observed and scanned using a Zeiss LSM 710 confocal microscope (Oberkochen, Germany) with lasers at 405 nm, and 543 nm wavelengths visualizing cell nuclei (DAPI) and reporter gene expression (mCherry), respectively.

### Data analysis

Data and statistical analyses were carried out in Microsoft Excel (part of Microsoft 365, version 18.2306.1061.0, Microsoft Corporation, Redmond, WA, USA) and Statistica 6.1 (StatSoft. Inc., Tulsa, OK, USA) softwares. An effect was determined significant if both the one-way or factorial ANOVA p-values and post hoc Tukey Honest Significant Difference (HSD) test p-values were smaller than 0.05. Ethics Approval The experiments were approved by the Animal Welfare Committee of the University of Pécs, and the National Scientific Ethical Committee on Animal Experimentation (ÁTET) at the Ministry of Agriculture (ethical license no.: BA02/2000-40/2021). All procedures fully complied with Decree No. 40/2013. (II. 14.) of the Hungarian Government and the EU Directive 2010/63/EU on the Protection of animals used for scientific purposes.

## Results

### Effect of exenatide on the food intake of control rats

**Figure 1:**
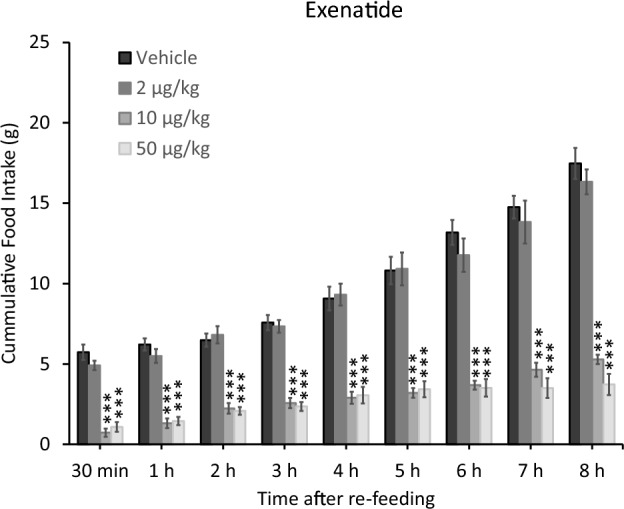
Effect of s.c. applied exenatide on the food intake of control rats. Post-hoc Tukey HSD tests: *** *p* < 0.001, compared to both the vehicle-treated and the 2 µg/kg exenatide-treated groups.

As a validation of our current food intake protocol, s.c. application of exenatide resulted in a robust food intake decrease at 10 and 50 µg/kg doses (Fig. 1). This effect was sustained and remained highly significant throughout the whole 8h post-re-feeding period. Statistical analysis yielded highly significant (*p* < 0.001) one-way ANOVA results if we compared the experimental groups against each other, in all time points, for example at the 30 min time point it was F_3,24_ = 56.729 (*p* < 0.001, Eta^2^ = 0.033), while at the end of the measurements at the 8h time point it remained significant, with a large effect size (F_3,24_ = 100.322, *p* < 0.001, Eta^2^ = 0.260). Post-hoc Tukey HSD analyses similarly revealed highly significant (*p* < 0.001) differences between the vehicle-treated and the low dose (2 µg/kg) exenatide vs. the 10 and 50 µg/kg exenatide groups, while there was no significant difference between the 10 and 50 µg/kg, nor the vehicle-treated and the low dose groups (Fig. 1).

### Effects of LH-hM4D(Gi) activation by CNO on the food intake of rats

**Figure 2:**
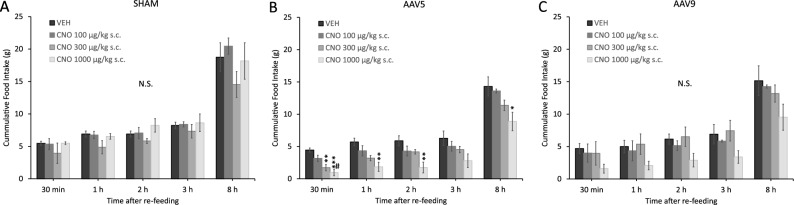
Effect of s.c. applied CNO stimulation on the food intake of LH-hM4D(Gi) rats. (**A**) Effect of CNO on SHAM-operated control rats; (**B**) Effect of CNO on AAV5 LH-hM4D(Gi) rats; (**C**) Effect of CNO on AAV9 LH-hM4D(Gi) rats. Post-hoc Tukey HSD tests: * *p* < 0.05, ** *p* < 0.01, *** *p* < 0.001, compared to the vehicle-treated group; #  *p* < 0.05, compared to the CNO 100 µg/kg group.

Subcutaneously applied CNO resulted in a significant food intake decrease only in the case of the AAV5 serotype, and only in the larger doses (300 and 1000 µg/kg, Fig. 2B), while in the case of the AAV9 serotype, the effects of CNO did not reach statistical significance (Fig. 2C). In detail, factorial ANOVA on DOSE and SEROTYPE, as the two main factors, yielded a significant effect for the SEROTYPE factor (F_10,48_ = 2.784, *p* < 0.01, Eta^2^ = 0.315), however neither the DOSE nor the interaction between SEROTYPE and DOSE were statistically significant. The subsequent one-way ANOVA tests showed significant differences in the case of AAV5, at 4 out of the 5 time points (30 min: F_3,11_ = 12.451, *p* < 0.001, Eta^2^ = 0.086; 1 h: F_3,11_ = 7.362, *p* < 0.01, Eta^2^ = 0.097; 2h: F_3,11_ = 6.134, *p* < 0.05, Eta^2^ = 0.106; 3h: F_3,11_ = 2.617, *p* = 0.104, Eta^2^ = 0.075; 8 h: F_3,11_ = 4.286, *p* < 0.05, Eta^2^ = 0.212), but were not statistically significant in the case of SHAM and AAV9 tests, at any time points. Post-hoc Tukey HSD analyses revealed the largest differences at the 30-min time point, while subsequently these differences either vanished, or their significance decreased (Fig. 2B).

### Effects of s.c. DCZ stimulation on the food intake of LH-hM4D(Gi) rats

**Figure 3:**
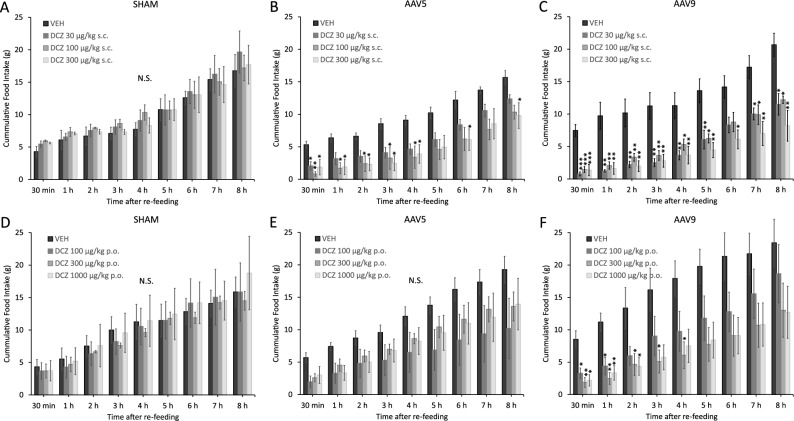
Effect of DCZ stimulation on the food intake of LH-hM4D(Gi) rats. (**A**): Effect of s.c. applied DCZ on SHAM-operated control rats; (**B**): Effect of s.c. applied DCZ on AAV5 LH-hM4D(Gi) rats; (**C**): Effect of s.c. applied DCZ on AAV9 LH-hM4D(Gi) rats; (**D**): Effect of p.o. applied DCZ on SHAM-operated control rats; (**E**): Effect of p.o. applied DCZ on AAV5 LH-hM4D(Gi) rats; (**F**): Effect of p.o. applied DCZ on AAV9 LH-hM4D(Gi) rats. Post-hoc Tukey HSD tests: * *p* < 0.05, ** *p* < 0.01, *** *p* < 0.001, compared to the vehicle-treated group.

Subcutaneously applied DCZ resulted in a strong, sustained and highly significant food intake decrease in both the AAV5 and the AAV9 serotype transfected animals (Fig. 3A–C). In detail, factorial ANOVA on the two key factors DOSE and SEROTYPE, showed significant effects for both (dose: F_27,71_ = 2.314, *p* < 0.01, Eta^2^ = 0.124; serotype: F_18,48_ = 2.103, *p* < 0.05, Eta^2^ = 0.246), nonetheless their interaction was not significant (*p* = 0.076). One-way ANOVA tests showed significant differences in the case of AAV5, at 8 out of the 9 food intake time points, while in the remaining time point (7h), a tendency was found to significant main effect (30 min: F_3,12_ = 6.858, *p* < 0.01, Eta^2^ = 0.035; 1h: F_3,12_ = 5.531, *p* < 0.05, Eta^2^ = 0.043; 2 h: F_3,12_ = 4.783, *p* < 0.05, Eta^2^ = 0.038; 3h: F_3,12_ = 5.311, *p* < 0.05, Eta^2^ = 0.068; 4 h: F_3,12_ = 4.494, *p* < 0.05, Eta^2^ = 0.063; 5 h: F_3,12_ = 3.597, *p* < 0.05, Eta^2^ = 0.062; 6 h: F_3,12_ = 3.972, *p* < 0.05, Eta^2^ = 0.076; 7 h: F_3,12_ = 3.187, *p* = 0.063, Eta^2^ = 0.067; 8 h: F_3,12_ = 4.364, *p* < 0.05, Eta^2^ = 0.066). In all time points with a significant main effect, the post-hoc analyses also showed significant difference between vehicle treatment and at least one DCZ dose, except of the 5h measurement, where post-hoc analysis only showed a tendency to significant effect of 100 and 300 µg/kg DCZ compared to vehicle treatment (*p* = 0.054 and *p* = 0.075, respectively). Furthermore, at 7h there was a tendency to significant effect of 100 µg/kg DCZ compared to vehicle treatment (*p* = 0.062).

Similarly, we found a significant DOSE main effect, in the case of AAV9, at all time points (30 min: F_3,12_ = 20.813, *p* < 0.001, Eta^2^ = 0.047; 1 h: F_3,12_ = 11.936, *p* < 0.001, Eta^2^ = 0.078; 2 h: F_3,12_ = 10.240, *p* < 0.01, Eta^2^ = 0.071; 3 h: F_3,12_ = 11.324, *p* < 0.001, Eta^2^ = 0.082; 4 h: F_3,12_ = 7.531, *p* < 0.01, Eta^2^ = 0.062; 5 h: F_3,12_ = 9.259, *p* < 0.01 Eta^2^ = 0.079; 6 h: F_3,12_ = 5.380, *p* < 0.05, Eta^2^ = 0.056; 7 h: F_3,12_ = 7.862, *p* < 0.01, Eta^2^ = 0.090; 8 h: F_3,12_ = 9.565, *p* < 0.01, Eta^2^ = 0.134), but there were no statistically significant effects in the SHAM group, at any time points. In AAV5 animals, post-hoc Tukey HSD analyses revealed the largest differences at the 30-min time point, while subsequently these differences alleviated, but overall the significant food intake effect of DCZ remained until the end of the 8h recording period (Fig. 3B). In the case of the AAV9 serotype transfection, the effects were even more robust (Fig. 3C), all the post-hoc Tukey tests showed *p* < 0.001-level significance at 30 min, moreover the effects of all doses of DCZ remained significant until the end of the 8h recording period.

### Effects of oral DCZ stimulation on the food intake of LH-hM4D(Gi) rats

Orally (*per os*, p.o.) applied DCZ resulted in much weaker effects compared to s.c. application (Fig. 3D–F), even against the fact that we applied one logarithmic step higher oral doses of DCZ (oral: 100–300–1000 µg/kg vs. s.c. 30–100–300 µg/kg). In detail, factorial ANOVA on DOSE and SEROTYPE × DOSE were significant (DOSE: F_27,62_ = 2.804, *p* < 0.001, Eta^2^ = 0.127; SEROTYPE × DOSE: F_54,112_ = 1.692, *p* < 0.05, Eta^2^ = 0.062). One-way ANOVA tests showed a significant food intake decrease in the case of AAV9, at the first 5 food intake measurement time points (30 min: F_3,10_ = 10.241, *p* < 0.01, Eta^2^ = 0.021; 1 h: F_3,10_ = 10.768, *p* < 0.01, Eta^2^ = 0.035; 2 h: F_3,10_ = 4.525, *p* < 0.05, Eta^2^ = 0.039; 3 h: F_3,10_ = 4.116, *p* < 0.05, Eta^2^ = 0.058; 4 h: F_3,10_ = 3.945, *p* < 0.05, Eta^2^ = 0.062), but DCZ was not effective in either the AAV5 or the SHAM groups at any time points. In AAV9 animals, post-hoc Tukey HSD analyses revealed the largest differences at the 30-min time point, while subsequently these differences disappeared from the 5h measurement time point and remained non-significant until the end of the 8h recording period. We would like to note that despite the absence of significant effects in the AAV5 group, there appears to be a non-significant, but strong tendency to reach an early effect at the 30- and 60-min time points, where both ANOVA and post-hoc tests yielded near-significant results (Fig. 3E; ANOVA 30 min: F_3,12_ = 3.102, *p* = 0.067; Post-hoc Tukey HSD test DCZ 100 µg/kg vs. VEH: *p* = 0.064; ANOVA 60 min: F_3,12_ = 3.044, *p* = 0.070; Post-hoc Tukey HSD test DCZ 100 µg/kg vs. VEH: *p* = 0.089).

### The timespan of the effect of DCZ

To further extend the functionality of our current rat food intake paradigm model, we tested the ability of the model to assess the timespan of effects of a selected actuator molecule. We chose DCZ with s.c. application in AAV5 LH-hM4D(Gi) rats, to test how long the food intake decreasing effect of DCZ last. We used the same food intake paradigm with a little modification: we applied different DCZ pre-treatment times (30 min, 1h, 3h, 16h before the reinstatement of food) and measured the 30-min food intake as the primary endpoint. We found that the pre-treatment time had a significant effect on the 30-min food intake (ANOVA: F_3,12_ = 9.078, *p* < 0.01), and consequently, post-hoc Tukey HSD analyses revealed that the 0.5h and the 1h pre-treatment times resulted in significantly reduced food intake compared to the effect caused by 16h DCZ pre-treatment (30 min.: 1.9 ± 0.4 g; 1 h: 2.3 ± 0.4 g; vs. 16h: 6.1 ± 0.4 g; *p* < 0.01 in both comparisons). Food intake at the 3h pre-treatment point did not significantly differ from any other time points (food intake at 3 h: 3.9 ± 1.0 g, N.S.), as it was numerically between the effect of the shorter (0.5–1 h) and the longer (16h) pre-treatment times.

### Histology

**Figure 4:**
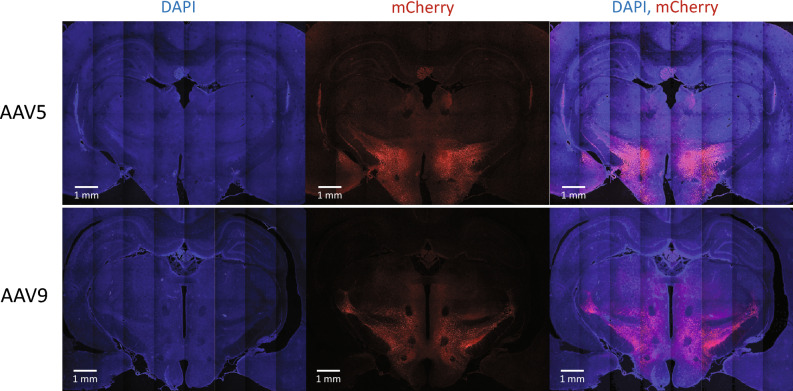
Histological examination of the expression of the transgene, containing the gene of mCherry fluorescent protein. The composite images indicate that the spreading of the viruses (AAV5, AAV9) mainly confined to the targeted lateral hypothalamus (LH) with some spreading in the neighbouring areas with the AAV5 serotype producing a more reliable labeling in the LH.

In the brains of all AAV-injected animals, an intensive expression of mCherry reporter protein was detected using confocal fluorescent microscopy (Fig. 4). The LH unambiguously expressed the reporter protein and therefore, confirmed the successful targeting and the sufficient DREADD receptor expression, regardless of the viral serotype. However, a somewhat larger area tended to be infected by AAV9 than AAV5. Furthermore, in both groups, the mCherry-labeled area appeared to confine adjacent brain regions outside the hypothalamus. Thus, in both groups, the zona incerta also showed the expression of the transgene, while in rats injected with AAV9, smaller parts of the ventral thalamus were also labeled with mCherry.

## Discussion

The overarching goal of our study was to develop a high translational value rodent behavioral assay, that supports the development of new chemogenetic systems in various CNS indications, offering a simple but robust in vivo efficacy readout to straightforwardly prove the effectiveness of any new chemogenetic constructs. Additionally, we were aiming to utilize the same assay to provide a simple way to estimate the basic pharmacokinetic properties of systemically applied actuator drugs.

We utilized exenatide as a food-intake-reducing reference drug to validate the human relevance of our model and to prove the translational power and predictability of our results. Exenatide is presumed to primarily promote weight loss in humans by reducing food consumption^23,24^, via acting as a mimetic of glucagon-like peptide 1 (GLP-1). Accordingly, the reported exenatide-induced robust food-intake-reducing effects (Fig. 1) validated the current preclinical model and proved its high translational value and human relevance.

We hereby demonstrated that several methodological parameters could affect the overall biological effectiveness of chemogenetic methods, including the serotype of the applied AAV viruses, the characteristics of the actuator drug, including its brain penetration, pharmacokinetic/pharmacodynamic properties, dose, and application route (i.e., oral or parenteral). From the currently investigated several parameters, we identified the AAV9 serotype, and DCZ with s.c. application, as the most effective combination, producing a robust food intake decrease in rats comparable to the effects of exenatide (Fig. 3C). Nevertheless, the AAV5 serotype, DCZ with oral application, and CNO were also shown to be significantly effective, but with a slightly weaker efficacy (Figs. 2 and Fig. 3). We also noticed that with all applied combinations of experimental parameters, the first 30-min food intake (after re-feeding) provided the greatest effect, therefore this seems to be the most ideal experimental endpoint. We also demonstrated that with different actuator pre-treatment times, the timespan of the effect of any actuator molecule can be conveniently assessed, and the acquired data can be used as an indirect pharmacokinetic estimation, that could be valuable when optimizing the effectiveness of newly developed actuator-receptor combinations, without the need of separate pharmacokinetic/pharmacodynamic studies. Finally, in terms of AAV serotype effectiveness, AAV9 seemed to provide a more robust hypothalamic (LH) and possibly some scattered extra-hypothalamic expression (Fig. 4) that seemed to correlate with its somewhat higher food intake decreasing potential. The somewhat larger area of expression in the case of AAV9 may be explained by its slightly larger virus particle titer in the applied injection volume. The AAV5 serotype, on the other hand, showed a slightly smaller, more focused LH infection, which, in turn, can possibly explain its marginally less efficacy. Nevertheless, a potential limitation of the present study was that the applied 1000 nl injection volume of viral vectors resulted in the expression of the transgene not only in the LH, but also in neighboring hypothalamic areas, the zona incerta and the subthalamic nucleus. Thus, to some extent, off-target effects might also influence the observed effects on food intake. If one would like to further explore the food intake or obesity-related effects of selective LH chemogenetic inhibition, it would be a logical next step to try to investigate the effectiveness of other virus serotypes (e.g., AAV2 or AAV8), and perhaps to try to reduce the affected brain area size by decreasing the applied virus volume (< 1000 nL) and/or the virus titer (< 10^1^^2^ vg/ml).

Taken these methodological considerations together, we have successfully developed, optimized, and validated an in vivo preclinical experimental model that is suitable for supporting various chemogenetic drug discovery projects. The present study provides a robust and reliable, but, at the same time, inexpensive and simple tool that can serve as a “first in vivo” model for characterizing the effectiveness of any newly developed CNS-targeted chemogenetic treatments. Moreover, we are presenting hereby the first human translatable evidence that CNS-targeted neuron-specific, however, subtype general chemogenetic stimulation can be similarly effective in reducing food intake than the currently most effective GLP-1 agonist pharmacological therapy available on the market in obesity indication^25^. Future studies are needed to validate that the currently reported food intake reduction can lead to sustained body weight and fat mass loss in a proper chronic disease model (e.g., in obese animals), and also could further investigate other potential chemogenetic target areas with similar human translatable chemogenetic approaches, such as the arcuate nucleus.

As a third major outcome of this study, we provided proof—in accordance with previous preclinical reports^7,8,26,27^—showing that DCZ is a more ideal chemogenetic actuator molecule for stimulating muscarinic DREADDs, compared to CNO, therefore we also do recommend a transition from the still widespread use of CNO to DCZ in all CNS-targeted preclinical studies using the muscarinic DREADD technology. We agree with the previous reports, that the higher efficacy and faster pharmacodynamic effect of DCZ can be explained by its better brain penetrance, better selectivity for the M3/M4 DREADDs, and its better metabolic stability, meaning that the effects of DCZ are not compromised by its metabolites with divergent pharmacological properties like in the case of CNO^5,28^.

Furthermore, the current results will strengthen the hypothesis that emerging innovative “genetic neuroengineering” techniques, like G-protein coupled DREADD chemogenetics, can be very promising potential future human therapeutic strategies, in general. This assumption is mostly based on the idea that chemogenetics can provide superior spatial targeting and selectivity, compared to conventional CNS pharmacotherapy methods. The only currently approved CNS therapeutic option, with similarly high spatial neuromodulatory ability is DBS, however, DBS needs chronically implanted devices that are permanently left in the brain, posing a considerable risk factor that might negatively influence the success of the otherwise highly effective method in the clinic^3^.

In conclusion, present research provides a high translational value preclinical evidence for the feasibility of LH chemogenetic modulation as a tool for CNS-targeted chemogenetic drug discovery, and simultaneously, it delivers the first translatable indirect in vivo POC for the therapeutic feasibility of chemogenetics in obesity indication. Based on our current results, we also support the hypothesis that future genetic neuroengineering treatments can offer highly effective alternative therapeutic options to both DBS and conventional pharmacotherapy in various CNS conditions, providing better spatial selectivity than pharmacotherapy, and offering a more tolerable approach for DBS-eligible patients. To aid this development, future studies must further explore the effectiveness (e.g., length of effect, viral serotype, titer, and dose dependence, etc.) and also the side-effect profile (e.g., potential neurotoxicity) of AAV-delivered CNS-targeted chemogenetic approaches in relevant preclinical disease models related to obesity.

## Data Availability

The authors declare that they make all experimental data available upon request to the corresponding author.
